# Expression of concern: An integrated salinity-driven workflow for rapid lipid enhancement in green microalgae for biodiesel application

**DOI:** 10.1039/d4ra90070g

**Published:** 2024-06-19

**Authors:** Gour Gopal Satpati, Prakash Chandra Gorain, Ishita Paul, Ruma Pal

**Affiliations:** a Phycology Laboratory, Department of Botany, University of Calcutta 35, Ballygunge Circular Road Kolkata-700019 West Bengal India rpalcu@rediffmail.com +91-033-2461-4849 +91-9433116320; b Agricultural and Food Engineering Department, Indian Institute of Technology Kharagpur-721302 India

## Abstract

Expression of concern for ‘An integrated salinity-driven workflow for rapid lipid enhancement in green microalgae for biodiesel application’ by Gour Gopal Satpati *et al.*, *RSC Adv.*, 2016, **6**, 112340–112355, https://doi.org/10.1039/C6RA23933A.

*RSC Advances* is publishing this expression of concern in order to alert readers that concerns have been raised over the integrity of the data published in this article.

Authors have reproduced images in **Fig. 3** without the appropriate referencing. **Fig. 4b1** and **Fig. 5b1** contain identical flow cytometry data for two different algae.

The authors were contacted for comment and asked to provide raw data but have not responded to these concerns. *RSC Advances* is publishing this expression of concern to alert readers to the concerns raised. An expression of concern will continue to be associated with the article until we receive conclusive evidence regarding the reliability of the reported data.

Laura Fisher

11th June 2024

Executive Editor, *RSC Advances*

